# The Profiles of Non-stationarity and Non-linearity in the Time Series of Resting-State Brain Networks

**DOI:** 10.3389/fnins.2020.00493

**Published:** 2020-06-11

**Authors:** Sihai Guan, Runzhou Jiang, Haikuo Bian, Jiajin Yuan, Peng Xu, Chun Meng, Bharat Biswal

**Affiliations:** ^1^MOE Key Laboratory for Neuroinformation, Center for Information in Medicine, School of Life Science and Technology, The Clinical Hospital of Chengdu Brain Science Institute, University of Electronic Science and Technology of China, Chengdu, China; ^2^The Laboratory for Affect Cognition and Regulation (ACRLAB), Key Laboratory of Cognition and Personality of Ministry of Education, Faculty of Psychology, Southwest University, Chongqing, China; ^3^Department of Biomedical Engineering, New Jersey Institute of Technology, Newark, NJ, United States

**Keywords:** resting-state fMRI, degree of stationarity, degree of non-linearity, test-retest, overlapping spatial

## Abstract

The linearity and stationarity of fMRI time series need to be understood due to their important roles in the choice of approach for brain network analysis. In this paper, we investigated the stationarity and linearity of resting-state fMRI (rs-fMRI) time-series data from the Midnight Scan Club datasets. The degree of stationarity (DS) and the degree of non-linearity (DN) were, respectively, estimated for the time series of all gray matter voxels. The similarity and difference between the DS and DN were assessed in terms of voxels and intrinsic brain networks, including the visual network, somatomotor network, dorsal attention network, ventral attention network, limbic network, frontoparietal network, and default-mode network. The test-retest scans were utilized to quantify the reliability of DS and DN. We found that DS and DN maps had overlapping spatial distribution. Meanwhile, the probability density estimate function of DS had a long tail, and that of DN had a more normal distribution. Specifically, stronger DS was present in the somatomotor, limbic, and ventral attention networks compared to other networks, and stronger DN was found in the somatomotor, visual, limbic, ventral attention, and default-mode networks. The percentage of overlapping voxels between DS and DN in different networks demonstrated a decreasing trend in the order default mode, ventral attention, somatomotor, frontoparietal, dorsal attention, visual, and limbic. Furthermore, the ICC values of DS were higher than those of DN. Our results suggest that different functional networks have distinct properties of non-stationarity and non-linearity owing to the complexity of rs-fMRI time series. Thus, caution should be taken when analyzing fMRI data (both resting-state and task-activation) using simplified models.

## Introduction

Functional magnetic resonance imaging (fMRI) has become an important method for investigating system-level brain activity (Biswal et al., 1995, 2010; He, 2013; Gordon et al., 2017; Gratton et al., 2018). The majority of fMRI research to date has used a simplified model based on the assumptions of stationarity and linearity (de Pasquale et al., 2010; Cabral et al., 2014; Panerai, 2014). Stationarity, in general, implies that the statistic or model parameter of interest does not change over time (Smith et al., 2012, 2013; Liu and Duyn, 2013; Allen et al., 2014; Shine et al., 2016; Suk et al., 2016; Yaesoubi et al., 2018). The stationarity assumption is also important for the frequency analysis of fMRI time series, as the Fourier transform is suitable for stationarity (Beck et al., 2006). Since resting-state fMRI (rs-fMRI) is a powerful tool for studying human functional brain networks, it is necessary to understand stationarity in the rs-fMRI time series. However, only a few studies have used fMRI signals to characterize the non-stationarity of time series. For example, Ou et al., used a Bayesian connectivity change point model to statistically investigate rs-fMRI signals and found that it could differentiate the temporal dynamics of functional interactions between children with attention deficit hyperactivity disorder and matched controls (Ou et al., 2014). Results from the study suggested that functional connectivity or interactions had temporally non-stationary characteristics. Muhei-Aldin and colleagues used non-parametric testing, i.e., the reverse arrangement test, to examine the stationarity of the fMRI signal during a motor sequence learning task and showed that the time series were non-stationary (Muhei-aldin et al., 2014). Bullmore et al., provided a review of wavelet methods used for the analysis of potentially non-stationary fMRI time-series signals (Bullmore et al., 2004).

Recently, several studies have investigated the temporal fluctuations in functional connectivity, i.e., dynamic functional connectivity, in the human brain and have interpreted their findings as evidence of non-stationary switching of discrete brain states (Allen et al., 2014; Hansen et al., 2015). Hutchison and colleagues used the rs-fMRI and sliding-window approach to study stimulus-independent fluctuations of functional connectivity within resting-state networks (Hutchison et al., 2013). They found that resting-state functional connectivity is not static and that resting-state networks can exhibit non-stationary spontaneous relationships irrespective of conscious and cognitive processing. Theoretically, the activity of neuronal assemblies should be non-stationary since it reflects the different stages of a self-organized process (Schoner and Kelso, 1988; Jin et al., 2017). However, several papers have reported contradictory findings regarding the non-stationarity in fMRI time series (Gaschler-Markefski et al., 1997; Hindriks et al., 2016; Laumann et al., 2017). For example, Gaschler-Markefski and colleagues reported that auditory tasks increased the non-stationarity in the fMRI time series of the auditory cortex (Gaschler-Markefski et al., 1997). Laumann et al., reported that the resting state condition yielded mean kurtosis measures closer to the stationary null model than task conditions, which seemed to suggest stationarity in the rs-fMRI signal (Laumann et al., 2017). Hindriks et al., found that the variation leading to dynamic functional connectivity was related to the length of the sliding window (Hindriks et al., 2016). To better understand the fMRI signal profile underlying functional connectivity, it is necessary to clarify whether the underlying processes are actually stationary or non-stationary (Thompson, 2018). Previous studies inferred non-stationarity in time series using task-related fMRI or based on the evidence of dynamic functional connectivity (Muhei-aldin et al., 2014; Ou et al., 2014). The quantitative non-stationarity profiles of rs-fMRI signals and various brain regions remain unclear.

On the other hand, the linear time-invariant (LTI) system plays a crucial role in modeling the fMRI time series to generate a transfer function from the stimulus to the neural output. The hemodynamic response used in fMRI data analysis is assumed to be a linear model in which the neuronal activity is thought to be linearly convolved with the underlying blood flow/volume (hemodynamic) changes (Esposito et al., 2002). While the fMRI time series approximates an LTI system for medium-duration stimuli, the fMRI response has been found to be non-linear for short-duration stimuli. For example, Wager et al., reported that the non-linearity of fMRI data may substantially influence the detection of task-related activations, particularly in rapid event-related designs when considering the non-linear effects on the response magnitude, onset time, and time to peak (Wager et al., 2005). Therefore, the presence of non-linear or deterministic behavior has been postulated in various physiological and pathological states (Freeman, 2000). Non-linearity postulates that irregular and seemingly unpredictable behaviors are not necessarily attributed to random external inputs to the systems but, on the contrary, can be the result of deterministic dynamical systems (Stam, 2005). Therefore, the detection of non-linearity is important and should be the first step before any non-linear analysis. Previous studies have shown the non-linear dynamics of brain activities by using EEG (Stam, 2005) and rs-fMRI (LaConte et al., 2004; Deshpande et al., 2006; Xie et al., 2008). For example, Xie et al., studied the spatiotemporal non-linear dynamics property in rs-fMRI signals of the human brain by using the spatiotemporal Lyapunov exponent analysis (Xie et al., 2008). Furthermore, the Delay Vector Variance (DVV) method has been used to characterize the non-linearity in fMRI time series (Freeman, 2003). Gultepe et al. used the DVV method to identify whether resting-state fMRI signals are linear or non-linear and found that the default-mode network had more linear signals compared to the visual, motor, and auditory networks, while the visual network had more non-linear signals than the others (Gultepe and He, 2013). Taken together, it is important to comprehensively study the degree of non-linearity of rs-fMRI time series in various large-scale brain networks and across whole-brain gray matter.

To probe the complexity and stability of a system such as the human brain, it is necessary to investigate both the non-linearity and stationarity of underlying dynamic activities given the inherent association and distinction between non-linearity and stationarity. In this study, we aim to comprehensively assess the profiles of non-stationarity and non-linearity in rs-fMRI time series for whole-brain gray matter voxels and functional networks. We compute quantitative measures for the degree of stationarity (DS) and the degree of non-linearity (DN) in nine healthy subjects with 10 test-retest rs-fMRI scans. We then calculate the test-retest reliability of DS and DN measures. We hypothesize that voxels and networks with stronger degrees of non-stationarity and non-linearity partially overlap and have varied test-retest reliability.

## Materials and Methods

### Data and Preprocessing

In total, 100 rs-fMRI scans were used in this study and were obtained from the Midnight Scan Club data (https://openneuro.org/datasets/ds000224/versions/1.0.0). Data were collected from 10 healthy, right-handed, young adult subjects (5 females and 5 males; age: 24–34 y) by using a Siemens Trio 3T MRI scanner over the course of 10 sessions conducted on separate days, each beginning at midnight. Within each session on 10 consecutive days, 30 min of rs-fMRI data were collected in which subjects visually fixated on a white crosshair presented against a black background. One subject (MSC08) was excluded due to the subject falling asleep during the scan, in line with the previous literature (Gordon et al., 2017). Therefore, the rs-fMRI data includes nine subjects, each with 10 sessions. The details about data acquisition and subject information have been reported previously (Gordon et al., 2017). Our data analysis included the following steps: (1) preprocess the rs-fMRI dataset; (2) calculate the DS of the preprocessed fMRI time series and create the network histogram map; (3) calculate the DN characterizations of the preprocessed fMRI time series and create the network histogram map; (4) determine the strength of DS and DN and identify their spatial overlap; (5) quantify the test-retest reliability of DS and DN.

The rs-fMRI preprocessing included the following: (1) discarding the first 10 volumes of each scan for signal equilibration, wherein subjects adapted to the environment; (2) slice time correction to account for temporal shifts in fMRI data acquisition; (3) correction for head motion; (4) use of the Friston-24 model to control head motion effects (Friston et al., 1996; Yan et al., 2013), followed by regressing out the signals from white matter and cerebrospinal fluid to reduce respiratory and cardiac effects (Fox and Raichle, 2007); (5) normalizing functional images into the standard MNI space by using the EPI template with the resampled voxel size of 4 mm; (6) spatially smoothing the result data using an 8-mm full width at half maximum (FWHM) Gaussian kernel; (7) band-pass filtering (0.009 Hz < *f* < 0.08 Hz); (8) extracting time series from whole-brain gray matter voxels and from functional networks based on Yeo's atlas (Yeo et al., 2011).

### The DS Characterization of fMRI Time Series

The Hilbert-Huang transform (HHT) is an adaptive time-frequency analysis method (Huang et al., 1998) and has been used to analyze non-linear and non-stationary signals (Qian et al., 2015). Compared to the sliding window approach, HHT can directly and quantitatively characterize the degree of stationarity in the time series. In addition, the HHT method has high performance in terms of both time-space and frequency-space resolution, which facilitates precise expressions of instantaneous frequency and is conducive to feature extraction of biomedical signals (Huang and Shen, 2005). The HHT mainly consists of two parts, namely the empirical mode decomposition (EMD) and the Hilbert transformation (Huang and Shen, 2005). The EMD is an efficient and adaptive method for extracting a set of intrinsic mode functions (IMFs) from non-linear and non-stationary time series (Lin and Zhu, 2012).

Signal *x*(*n*) of length *N* can be decomposed by EMD to obtain *M* basic mode components *c*_1_, *c*_2_, ⋯ , *c*_*M*_ and residual component*r*_*M*_.

(1) $$\begin{array}{r} x\left(n\right)=\overset{M}{\underset{j=1}{\sum }}c_{j}+r_{M} \end{array}$$

For each of the IMFs, using Hilbert transform, we obtain

(2) $$\begin{array}{r} x\left(n\right)=\overset{M}{\underset{j=1}{\sum }}a_{j}\left(n\right)e^{iw_{j}\left(n\right)n} \end{array}$$

The Hilbert spectrum of *x*(*n*) can thus be expressed as:

(3) $$\begin{array}{r} H\left(w,n\right)=\sum {b_{j}a}_{j}\left(n\right)e^{iw_{j}\left(n\right)n} \end{array}$$

where

(4) $$\begin{array}{l} b_{j}=\{\begin{array}{c} 1\;w_{j}=w\\ 0\;other \end{array} \end{array}$$

The boundary Hilbert spectrum of *x*(*n*) is

(5) $$\begin{array}{r} h\left(w\right)=\overset{N-1}{\underset{n=0}{\sum }}H\left(w,n\right) \end{array}$$

The average boundary spectrum is defined as

(6) $$\begin{array}{r} B\left(w\right)=\frac{1}{N}h\left(w\right) \end{array}$$

Thus, the DS can be defined as

(7) $$\begin{array}{r} DS\left(w\right)=\frac{1}{N}\overset{N-1}{\underset{n=0}{\sum }}{\left(1-\frac{H\left(w,n\right)}{B\left(w\right)}\right)}^{2} \end{array}$$

*DS*(*w*) is capable of quantitatively detecting the stationarity of the data. For the stationarity process, the Hilbert spectrum does not change with time; it only contains the horizontal contour, that is *DS*(*w*) = 0. If the Hilbert spectrum is time-dependent, then *DS*(*w*) > 0, and as *DS*(*w*) increases, the signal's non-stationarity is enhanced.

### The DN Characterization of fMRI Time Series

The DVV method characterizes a time series based upon its predictability and compares the result to those obtained for linearized versions of the signal (surrogates) (Gautama et al., 2004). Based on a set of *N* delay vectors (DVs), denoted by *x*(*k*) = [*x*_*k*−*m*_, *x*_*k*−*m*+1, …_, *x*_*k*−1_], a vector containing *m* consecutive time samples. Every DV *x*(*k*) has a corresponding target, namely the following sample *x*_*k*_. For a given embedding dimension *m*, the mean target variance, σ^*2^, is computed over all sets Ω_*k*_. A set Ω_*k*_ is generated by grouping those DVs that are within a certain distance from *x*(*k*), which is varied in a manner standardized with respect to the distribution of pairwise distances between DVs. This way, the threshold automatically scales with the embedding dimension *m*, as well as with the dynamical range of the time series at hand, and thus, the complete range of pairwise distances is examined. The proposed DVV method can be summarized as follows for a given embedding dimension *m*:

- The mean, μ_*d*_, and standard deviation, σ_*d*_, are computed over all pairwise distances between DVs, ||*x*(*i*) − *x*(*j*) ||(*i* ≠ *j*).
- The sets Ω_*k*_ are generated such that Ω*n* = {*x*(*i*)|||*x*(*k*) − *x*(*i*) || ≤ τ_*d*_}, i.e., sets that consist of all DVs that lie closer to *x*(*k*) than a certain distance τ_*d*_, taken from the interval [*min*{0, μ_*d*_ − *n*_*d*_σ_*d*_}], e.g., uniformly spaced, where *n*_*d*_ is a parameter controlling the span over which to perform the DVV analysis.
- For every set Ω_*k*_, the variance of the corresponding targets, $\sigma_{k}^{2}$, is computed. The average over all sets Ω_*k*_, normalized by the variance of the time series, $\sigma_{x}^{2}$, yields the measure of unpredictability, σ^*^2:

(8) $$\begin{array}{r} \sigma^{{}^{*}2}=\frac{1}{N}\overset{N}{\underset{k=1}{\sum }}c_{j}\sigma_{k}^{2}\sigma_{x}^{2} \end{array}$$

The deviation from the bisector line is thus an indication of non-linearity and can be quantified by the root mean square error (RMSE) between the σ^*2^'s of the original time series and the σ^*2^'s averaged over the DVV plots of the surrogate time series (note that while computing this average, as well as with computing the RMSE, only the valid measurements are taken into account, and then the DN is obtained). In this way, a single test statistic is obtained, and traditional (right-tailed) surrogate testing can be performed (the deviation from the average is computed for the original and surrogate time series).

### Threshold for Strong DS and DN

**Figure 1:**
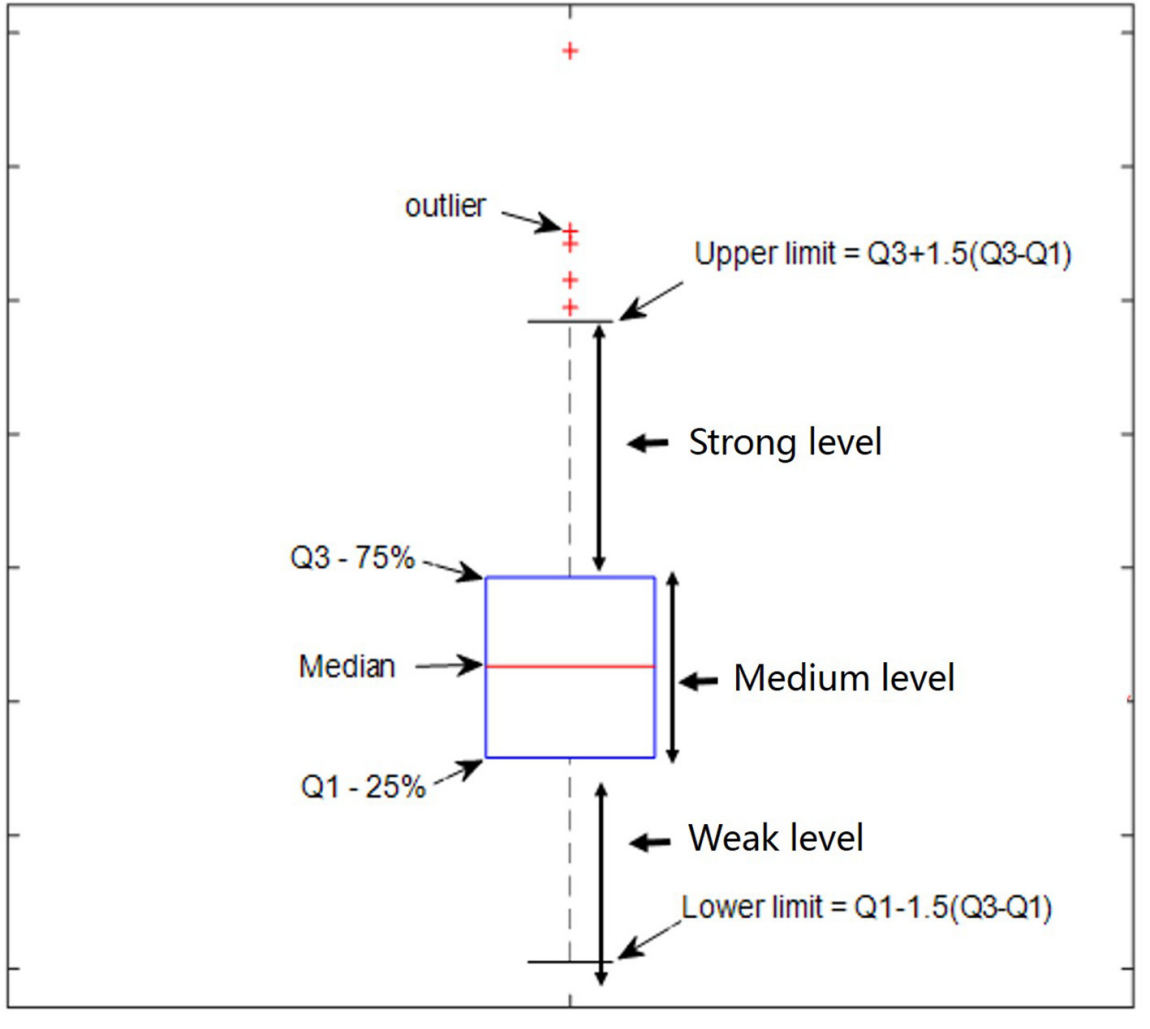
Defined percentage ratio by using the quartile method.

The quartile method (Hyndman and Fan, 1996), as shown in Figure 1, was used to determine the relative thresholds for strong DS and DN, which made use of the whole-brain DS and DN values. The quartile is a numerical value obtained when all values are arranged from small to large in statistics and are divided into four equal positions. The third quartile was arbitrarily selected as the threshold for strong DS and DN in this study.

Definition

(9) $$\{\begin{array}{c} Q_{lower\;limit}<value\;DS\leq Q_{1}\;weak\;level\;non\text{-}stationary\\ Q_{1}<value\;DS\leq Q_{3}\;medium\;level\;non\text{-}stationary\\ Q_{3}<value\;DS\leq \;Q_{upper\;limit}\;strong\;level\;non\text{-}stationary \end{array}$$

(10) $$\{\begin{array}{c} Q_{lower\;limit}<value\;DN\leq Q_{1}\;weak\;level\;non\text{-}linearity\\ \;Q_{1}<value\;DN\leq Q_{3}\;medium\;level\;non\text{-}linearity\\ Q_{3}<value\;DN\leq \;Q_{upper\;limit}\;strong\;level\;non\text{-}linearity \end{array}$$

Where

(11) $$\begin{array}{r} Q_{upper\;limit}=Q_{3}+1.5\times \left(Q_{3}-Q_{1}\right) \end{array}$$

(12) $$\begin{array}{r} Q_{lower\;limit}=Q_{1}-1.5\times \left(Q_{3}-Q_{1}\right) \end{array}$$

### Histogram Map and Overlap Map

After calculating voxel-based values of DS and DN within the gray matter mask, 90 maps were obtained for nine subjects and their 10 test-retests. Resulting maps were combined to identify the histogram map of DS and DN and the distribution of strong DS and DN as well as the overlap and distinctions by using an *a priori* functional network atlas (Yeo et al., 2011) (including VN: visual network, SMN: somatomotor network, DAN: dorsal attention network, VAN: ventral attention network, LIMB: limbic network, FPN: frontoparietal network, and DMN: default-mode network). Finally, the percentage of overlapping voxels for each network was calculated.

### Test-Retest Reliability

Test-retest studies are essential to determine the reliability of rs-fMRI measures (Noble et al., 2019). To evaluate the test-retest reliability as well as the within- and between-subjects variability of DS and DN, we computed the intraclass correlation (ICC) (Shrout and Fleiss, 1979) and obtained the test-retest reliability maps for DS and DN.

(13) $$\begin{array}{r} \text{ICC}=\frac{\text{BMS}-\text{EMS}}{\text{BMS}+\left(k-1\right)\text{EMS}} \end{array}$$

Equation (13) estimates the correlation of the subject signal intensities between sessions, modeled by a two-way analysis of variance, with random subject effects and fixed session effects. In this model, BMS is between-targets mean square, EMS is error sums of squares, and *k* is the number of repeated sessions. For statistical evaluations, a significance threshold of *p* < 0.05 was used.

### Similarity Analysis

To explore the similarity of DS or DN between functional networks, Pearson's correlation was conducted across subjects and sessions by using average values within each network. Furthermore, we explored the similarity between DS and DN within 10 axial slices. Average values of DS and DN across subjects and sessions were correlated for each slice.

## Results

### Distribution of DS and DN in Terms of Voxels and Networks

**Figure 2:**
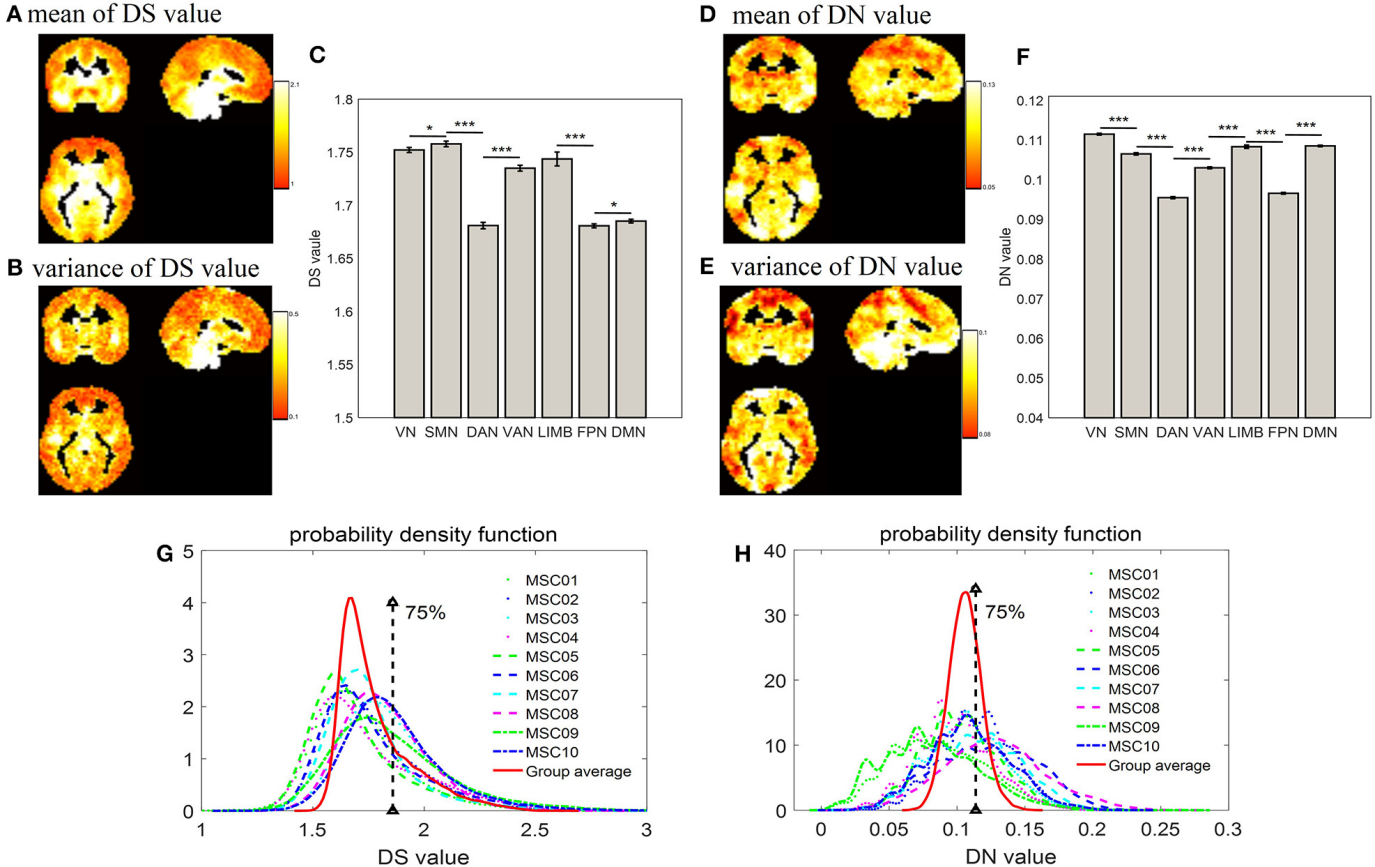
Distribution of DS and DN in terms of voxels and networks. **(A)** and **(D)** show spatial distribution of group average DS and DN in terms of voxels across nine subjects and their 10 test-retest sessions; **(B,E)** show variance of spatial distribution of DS and DN in terms of voxels; **(C,F)** show bar plot of DS and DN, respectively, within intrinsic brain networks according to a priori functional network atlas (Yeo et al., 2011); **(G,H)** show probability density estimates of voxel-wise DS and DN values for each subject and group average, respectively; 75% is the strong threshold level, which is obtained by way of the quartile method (in Figure 1). The two-sample *t*-test showed the significant differences between each other (*p < 0.05, ***p < 0.001).

As displayed by three slice maps in terms of voxels across nine subjects and their 10 test-retest sessions, we found that the resting-state brain had varied DS and DN values in different regions (Figures 2A–E). Mean value maps were plotted by using the average values of DS and DN across subjects and sessions, respectively (Figures 2A,D). The variance value maps were plotted by using the variance values of DS and DN across subjects and sessions, respectively (Figures 2B,E). Although DS and DN largely shared the same regions, they still had their own unique distribution. For example, the peak intensity within seven networks differed between DS and DN (Figures 2C,F). For DS, the SMN (DS = 1.758 ± 0.00270) is composed of relatively higher non-stationary signals compared to the VN (DS = 1.752 ± 0.00240) and DAN (DS = 1.681 ± 0.00300) resting-state network time series, and the *p*-values from the two-sample *t*-tests are *p* = 0.031 and *p* = 0, respectively; LIMB (DS = 1.744 ± 0.00650) is composed of relatively higher non-stationary signals compared to the FPN (DS = 1.681 ± 0.00190) and DMN (DS = 1.685 ± 0.00170), and the p-values in the corresponding two-sample *t*-tests are both *p* = 0; VAN (DS = 1.735 ± 0.00280) has relatively higher non-stationarity signals than DAN (DS = 1.681 ± 0.00300), and the *p*-value in the two-sample *t*-test is *p* = 0. In addition, for DN, it was shown that the DAN (DN = 0.0955 ± 0.00026) has lower non-linearity relative to the SMN (DN = 0.1065 ± 0.00029), VN (DN = 0.1115 ± 0.00022), LIMB (DN = 0.1083 ± 0.00041), and VAN (DN = 0.103 ± 0.00024), and all the corresponding *p*-values from the two-sample *t*-tests are *p* = 0. The DMN (DN=0.1085 ± 0.00019) is composed of relatively higher non-linear signals compared to the FPN (DN=0.0966 ± 0.00022) resting-state network time series, and the *p*-value in the two-sample *t*-test is *p* = 0. Also, Figures 2G,H show a probability density estimate for voxel-wise DS and DN values for each subject and group average made by using ksdensity.m in MATLAB. In Figures 2G,H, 75% is the strong threshold level; we can use the quartile method (in Figure 1) to get it. As shown in Figures 2G,H, the statistical characteristics of DS were different from those of DN, in that DS has a long tail, while DN has tails more similar to a normal distribution (Figures 2G,H).

### Distribution of Strong DS and DN in Terms of Voxels and Networks

**Figure 3:**
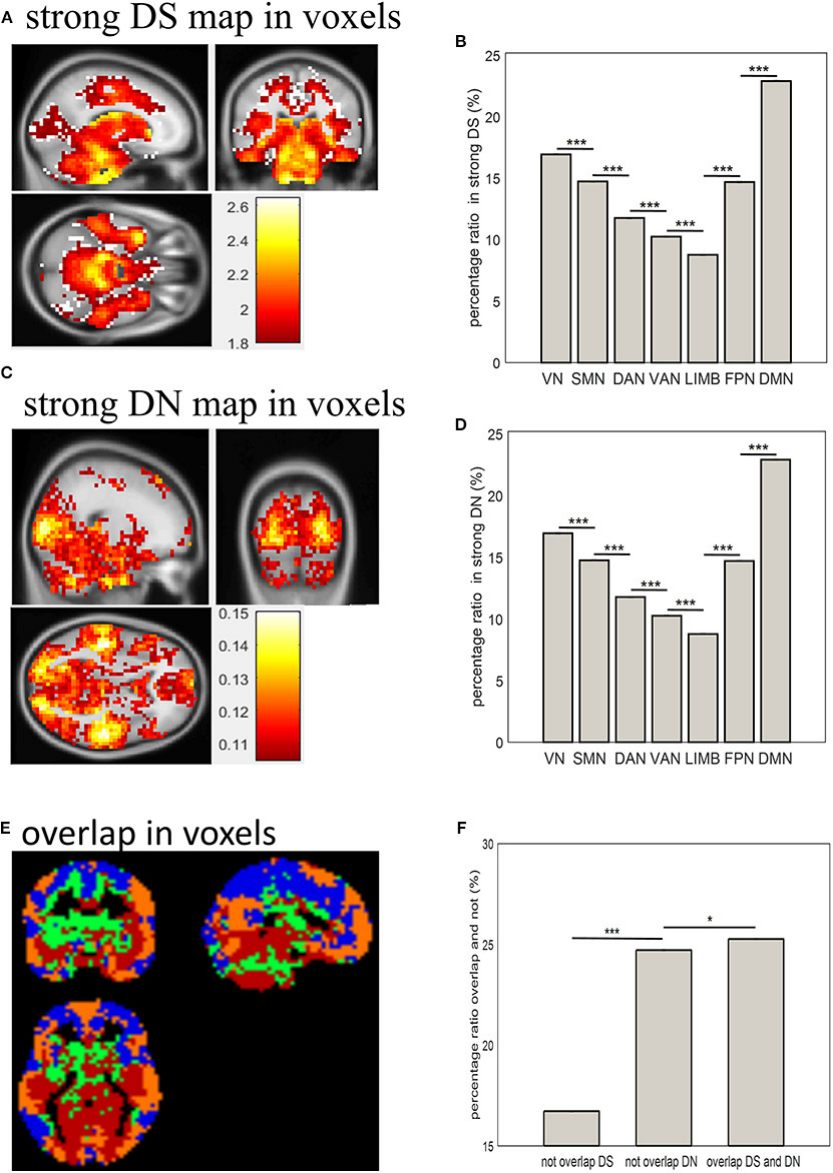
Distribution of strong DS and DN in terms of voxels and networks. **(A,C)** show spatial distribution of strong DS and DN in terms of voxels; **(B,D)** show bar plots of percentage ratios of voxels of DS and DN, respectively, for seven networks; **(E)** shows spatial distribution of overlapping strong DS and DN in terms of voxels: blue indicates brain regions with weak DS and DN (below 75%); green and yellow indicated unique brain regions with strong DS and DN (above 75%) respectively; red indicates brain regions with strong overlapping DS and DN (above 75%); **(F)** shows a bar plot of the percentage ratios of voxels overlapping and not overlapping between strong DS and DN. Two-sample *t*-tests showed significant differences between them (*p < 0.05, ***p < 0.001).

Combining Figure 1 and the distribution of DS and DN in terms of voxels (Figure 2) allows the distribution of strong DS and DN maps in the whole brain (shown in Figures 3A,C) to be obtained. The overlap maps between strong DS and strong DN are shown in Figure 3E. We found that the same regions exist in both DS and DN, but each has its own unique distribution. The histogram maps of percentage ratios of voxels of the DS and DN characterized networks are shown in Figures 3B,D, respectively, and we found that the percentage ratio of voxels for each network ranked from largest to smallest was: DMN, VN, SMN, FPN, DAN, VAN, and LIMB. Based on Figure 3F, the percentage ratio of overlap and non-overlap ranked from largest to smallest was overlapping DS and DN (25.72%), non-overlapping DN (24.72%), and non-overlapping DS (16.73%).

### Test-Retest Reliability for DS and DN

**Figure 4:**
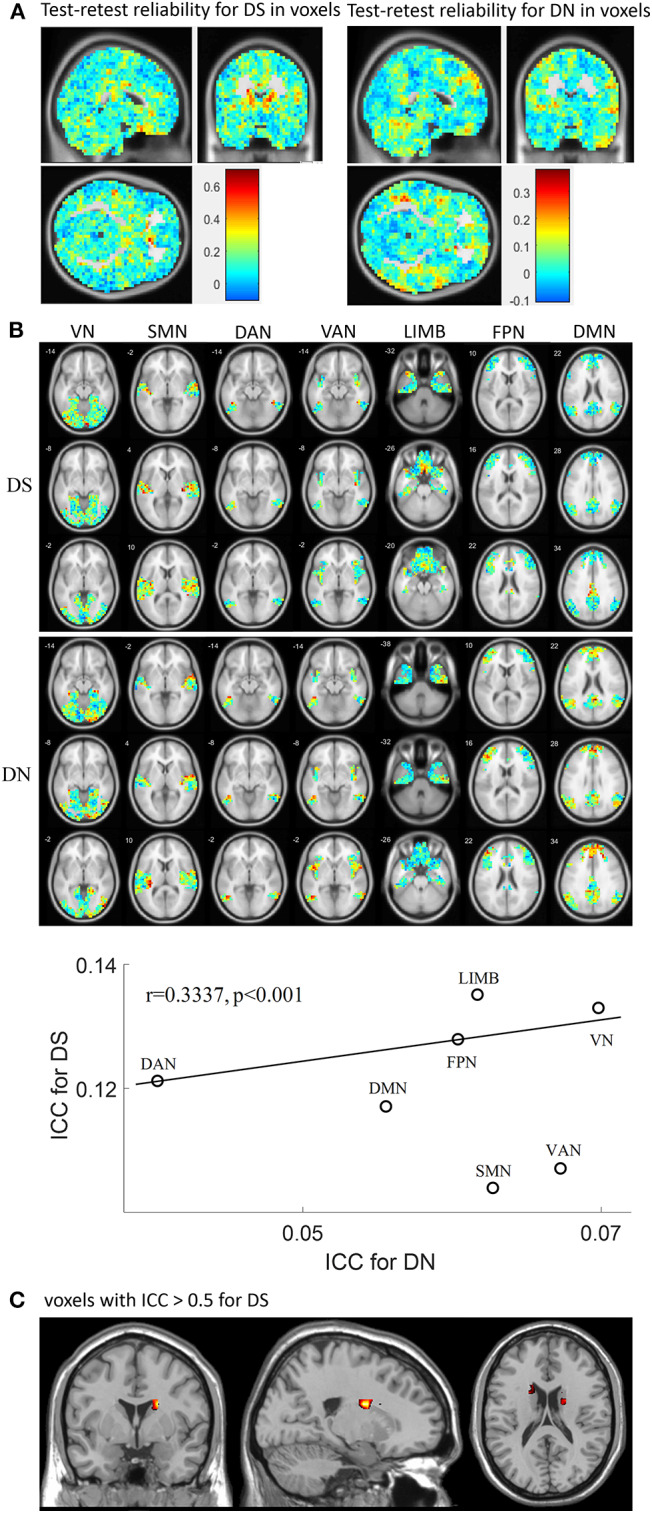
Test-retest reliability for DS and DN. **(A)** shows spatial distribution of test-retest reliability for DS and DN, respectively, in terms of voxels calculated by using different session numbers; **(B)** ICC maps from the DS (first row) and DN (second row) with networks: VN SMN DAN VAN, LIMB, FPN, and DMN; in the third row, the correlation between ICC for DN and ICC for DS is displayed. **(C)** ICC maps from the DS when ICC ≥ 0.5.

Test-retest reliability for DS and DN was analyzed in terms of voxels and networks (Figure 4) by using all rs-fMRI data. First, the spatial distribution of test-retest reliability for DS and DN in terms of voxels as plotted, as shown in Figure 4A, and the ICC values of DS were found to be higher than those of DN. Then, test-retest reliability for DS and DN were analyzed in terms of networks, as presented in Figure 4B, which shows ICC maps from the DS and DN with networks: VN, SMN, DAN VAN, LIMB, FPN, and DMN. From Figures 4A,B, we found that the ICC values of DS and DN for each network were different and also found that the ICC values of DS were higher than those of DN in each network. Furthermore, upon inspecting Figures 4A,B, it was found that most of the voxels still have ICCs hovering around 0.2–0.3. Each network demonstrated lower ICC for DN and DS, while DS and DN displayed significant correlation (correlation coefficient *r* = 0.3337, *p* < 0.001) across voxels and networks as calculated by using the cftool.m in MATLAB. The spatial distribution of test-retest reliability for DS in terms of voxels when ICC ≥0.5 was plotted in Figure 4C. From Figure 4C, voxels reaching an ICC of at least 0.5 were mainly found on the DMN, FPN, LIMB, and VAN. More specific to the brain regions, there were also some voxels with ICC >0.5, such as Tempor_Pole_Sup_R (X = 34, Y = 6, Z = −24), Caudate_L (X = −18, Y = −6, Z = 24), Caudate_R (X = 18, Y = 6, Z = 20), Cingulum_Mid_R (X = 6, Y = −20, Z = 40), Rectus_R (X = −2, Y = 18, Z = −20), and Congulum_Ant_L (X = 2, Y = 30, Z = 0).

### Similarity Analysis for DS and DN

**Figure 5:**
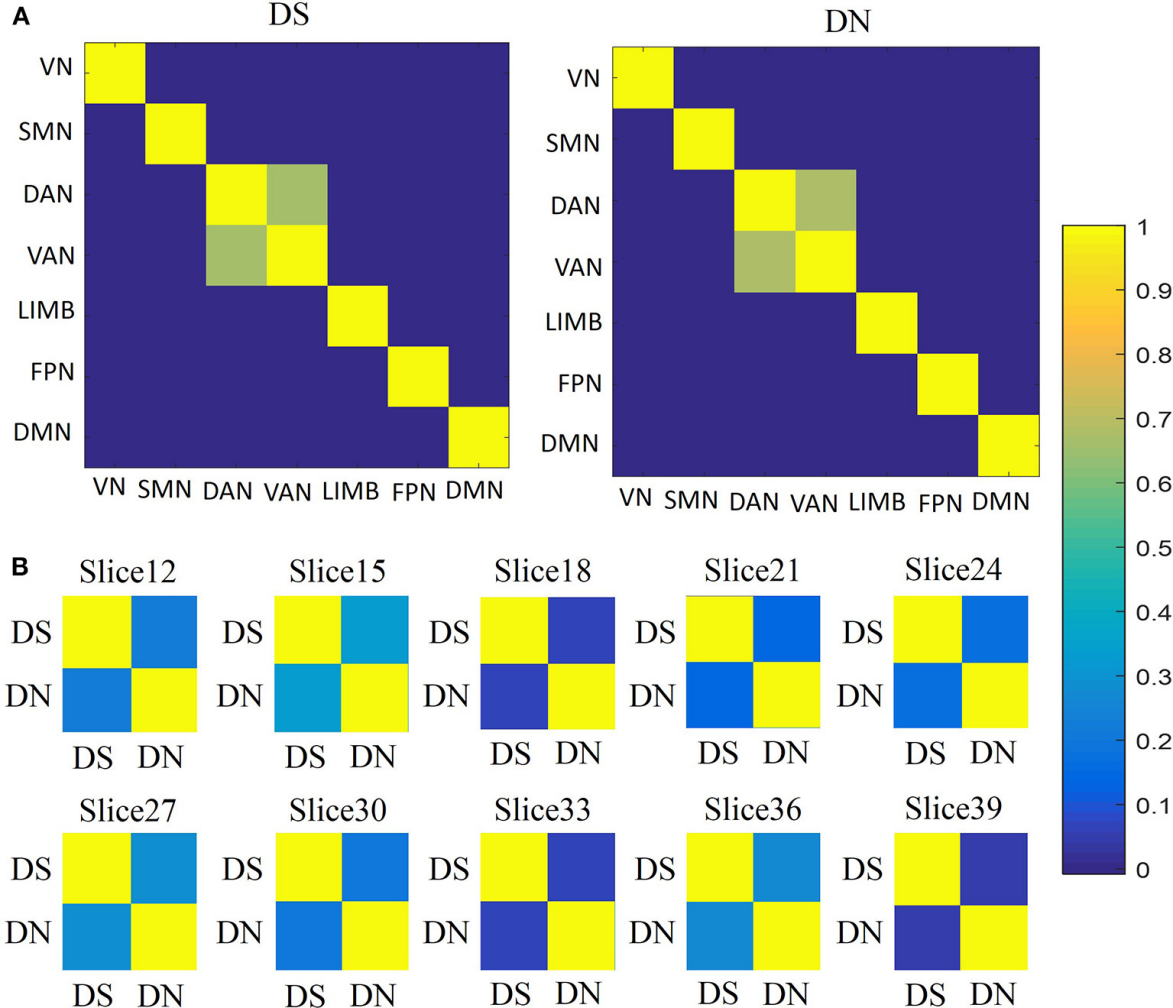
Similarities analysis. **(A)** Between network similarities, using the average values of DS and DN within networks, across subjects and sessions by using Pearson's correlation. Correlation matrices for the DS (first column) and DN (second column) associated with networks: VN, SMN, DAN, VAN, LIMB, FPN, and DMN. **(B)** The similarities between DS and DN for each slice, across subjects and sessions, according to Pearson's correlation.

For the similarity between networks (Figure 5A), we found that DAN and VAN were correlated for DS and DN (*r* = 0.6559, *p* = 0), whereas the association was not significant for the other five networks (Figure 4A). Furthermore, we used correlation matrices for the DS and DN associated with different spatial brain slices (Figure 5B). The slice-based similarity analysis showed a low correlation between DS and DN (*r* = 0.3400), which varied in different slices. High correlation corresponds to the similarity of the DS and DN in intra-slice variability and the correlation coefficients are different in different slices.

## Discussion

The human brain is a complex system, and there has been growing research interest in analyzing the complex brain networks by using rs-fMRI time series (Fox and Raichle, 2007; Biswal et al., 2010; de Pasquale et al., 2010; Liu and Duyn, 2013; Gao et al., 2018). In this work, the non-stationarity and non-linearity in rs-fMRI data of the human brain were detected by using the DS and DN measures. We quantified the degrees of non-stationarity and non-linearity in the time series of rs-fMRI by using the HHT and DVV methods. DS and DN were computed in terms of voxels across nine subjects and for their 10 test-retest sessions. We found that DS and DN had overlapped spatial distributions together with varied characteristics across typical intrinsic brain networks, as well as different test-retest reliabilities. The DS and DN characterization of the rs-fMRI time series analysis has provided a new method of analyzing ongoing activities within the resting-state brain.

### Distribution of DS

In this study, voxel-based and network-specific DS were examined. The mean DS value ranged from 0.1 to 2.1 over the whole brain, with the higher DS values in the brainstem, thalamus, striatum, temporal and occipital cortex, and cerebellum (Figure 2A), as well as in the networks SMN, VN, LIMB, and VAN (Figure 2B). From a theoretical point of view, the activity of neuronal assemblies should be non-stationary since it reflects the different stages of a self-organized process (Schoner and Kelso, 1988; Jin et al., 2017). A previous study revealed by analyzing EEG signals that brain activity is essentially non-stationary (Kaplan et al., 2005). An fMRI study also confirmed that there was non-stationary brain activity during an auditory task (Gaschler-Markefski et al., 1997). Using rs-fMRI, dynamic functional connectivity has been researched to delineate the non-stationary changes in brain activity synchronization (Xie et al., 2008; Ou et al., 2014). However, a recent study revealed that it is difficult to detect the non-stationarity in a typical rs-fMRI scan of 10 min using the sliding window approach because the effect of non-stationarity detection varies with the amount of data. Therefore, the authors pointed out that it is not optimal to use the sliding window approach for non-stationarity analysis (Hindriks et al., 2016). Using the HHT method, the current study demonstrated non-stationary signal fluctuation in widespread brain regions and functional networks, which confirmed the non-stationarity in the rs-fMRI signal and provides a quantitative DS map.

### Distribution of DN

Using the DVV method, we found that voxels with strong DN are spatially distributed across different functional networks. From the DN value, the DAN showed a lower non-linear signal, and the VN, DMN, LIMB, and SMN showed higher non-linear signals (Figure 2F). The ranking of the DN value for each network from largest to smallest is as follows: VN, DMN, LIMB, SMN, VAN, FPN, and DAN. This suggests that despite the absence of external stimuli to VN, DMN, and LIMB, the baseline activity of those networks may be more complex than that of other systems. Both Gautama and Mandic have shown that the default-mode resting-state network time series is relatively more linear than time series in the auditory and motor networks (Gautama et al., 2003; Mandic et al., 2008). Gultepe and He previously reported that visual networks were more non-linear than the motor and auditory systems (Gultepe and He, 2013). Our finding supported the conclusion that without external stimulus, during resting state, the baseline activity of the visual cortex is more complex than the motor and auditory systems, which may be associated with complex functional organization for visual processing. Gultepe and He (2013) showed that in a task-based study using macaque BOLD and monocrystalline iron oxide particle (MION) signals the recruitment of physiological inputs such as cerebral blood volume, flow, and metabolic rate of oxygen into these two systems may be increased compared to in a resting state study where there is no task (Gautama et al., 2003). This may reflect their conclusion that the BOLD signal is more non-linear than the MION signal, which depends on fewer physiological parameters. The lower embedding dimension may be indicative of the lower complexity of resting-state systems within the brain; it is necessary to choose dimensions high enough to capture the phase space of the dynamical system (Gautama et al., 2004).

### Overlap Between DS and DN

From the overlap of the spatial distributions of strong DS and DN in terms of voxels, the percentage ratio of voxels overlapping between strong DS and DN values was 25.72% and was relatively high compared to unique regions of strong DN (that is, those regions that have strong DN and weaker DS properties) (24.72%) and unique regions of strong DS (those regions that have strong DS and weaker DN properties) (16.73%). This suggests that regions that overlap between DS and DN exist but that each has its own unique distribution. Both stationarity and linearity can be determined by the complexity and stability of the activities of brain regions, making them inseparable. The two indicators reflect the profile of stationary time series and linear system, respectively, which have their own unique characteristics. For example, the larger the DS value in the fMRI signals, the more complex brain activities will be, while the larger the DN value, the more unstable brain activities will be. Thus, overlapping of strong DN and strong DS in certain regions demonstrated that those brain regions have simultaneous non-stationary and non-linear signals. The DMN showed the largest percentage ratio of voxels with strong DN and DS values. Thus, among the overlapping regions of strong DN and DS, DMN was the largest. This suggests that the DMN has both non-stationary and non-linear signals. It has been hypothesized that the activity of the DMN is related to spontaneous thoughts, i.e., intrinsic attention/information processing (Raichle et al., 2001). The DMN has been observed to be active at rest and deactivated during active task-states (Damoiseaux et al., 2008). In addition, the existence of unique regions of strong DN (24.72%) and unique regions of strong DS (16.73%) revealed that there are also some regions with their own unique characteristics, such as those with more complex and more stable brain activities, contrasting with those with simpler and more unstable brain activities. This further demonstrates that the brain is a complex system.

### Test-Retest Reliability and Similarity Analysis

We inferred that the ICC values of DS were larger than those of DN in terms of voxels and networks and that the ICC values of DS and DN for each network were different. DS and DN exhibited significant correlation across voxels and networks for each network. Moreover, the test-retest reliability values for DS and DN across 10 sessions were surprisingly low because most voxels had ICC values below 0.5. Most of the voxels had ICCs hovering around 0.2–0.3, while a few voxels reached 0.6, and voxels reaching an ICC of at least 0.5 were mainly found on certain brain regions, such as Tempor_Pole_Sup_R (X = 34, Y = 6, Z = −24), Caudate_L (X = −18, Y = −6, Z = 24), Caudate_R (X = 18, Y = 6, Z = 20), Cingulum_Mid_R (X = 6, Y = −20, Z = 40), Rectus_R (X = −2, Y = 18, Z = −20), and Congulum_Ant_L (X = 2, Y = 30, Z = 0); the ICC values in other brain regions were no larger than 0.5. These results provide a quantitative basis for the test-retest reliability of non-stationarity or non-linearity. In terms of similarity between networks, DAN and VAN were correlated for DS and DN, while the association was not significant for other networks. Thus, DS and DN can be recognized to have a good ability to predict network types. Moreover, the slice-based similarity analysis showed low correlation between DS and DN, which varied in different slices. High correlation corresponds to similarity of the DS and DN in intra-slice variability, and the correlation coefficients are different in different slices. The main reason for this is that the percentage ratio of overlapping voxels with strong DS and DN values was 25.72%. The overall reliability of topological measures was similar to that of other parameters derived from rs-fMRI, such as correlation significance, correlation valence (positive vs. negative correlations), and network membership (Shehzad et al., 2009). Resting-state data itself is a complex aggregation of different brain networks whose activity profiles overlap (Greicius et al., 2003), but this is also so in brain states that reflect cognitive and emotional processing (Damoiseaux et al., 2006).

Previous works have suggested that the fMRI signal consists of non-linear and non-stationary components (Xie et al., 2008; Ou et al., 2014), but these components have often been discarded in conventional generalized linear modeling and functional connectivity (analysis based on Pearson's correlation). In this study, we introduced a quantitative statistical method to identify the scale of non-linearity and non-stationarity in fMRI signals. The DS and DN measures enable the characterization of not only the brain's signal properties across specific regions but also the individual subject's brain dynamic features. Future fMRI research should compute DS and DN as part of the quality control step for preprocessing as indicators of data quality, particularly when dealing with cross-sectional comparisons. For example, individual DS and DN values should be identified for clinical populations and healthy controls, respectively, and then controlled as covariates in the group comparison of their functional connectivity. In the study of dynamic functional connectivity associated with non-stationary features, future research should investigate the potential relationship between dynamic measures and both DS and DN. It is also worth understanding the alterations of DS and DN linked with fMRI preprocessing, such as the complex influence of micromovement on fMRI signals. Taken together, the current study revealed that the quantitative map of the whole-brain DS/DN will provide a tool for future research to further explore the effect of DS/DN on fMRI measures such as functional connectivity.

## Limitations

The present work has several potential limitations worth considering. In this paper, we estimated non-stationarity/non-linearity effects, respectively. Our major findings showed that these non-stationarity/non-linearity effects varied across different functional networks. One potential limitation was that this work focused on rs-fMRI signals and thus did not provide DS/DN measures based on task-fMRI data, although several previous studies have pointed out that non-stationarity/non-linear effects may differ among different tasks (Wager et al., 2005; Muhei-aldin et al., 2014; Ou et al., 2014). The second potential limitation of this study is that this work only focused on voxels and seven functional networks from Yeo's functional network atlas (Yeo et al., 2011). Depending on the parcellation number of functional networks (up to hundreds), the corresponding ICC may be different. The spatial extent of a region and how it may affect the ICC should be carefully investigated. The third limitation might be the imaging length of 30 min used in this study, which might affect the DS/DN. Previous studies have shown the ICC of functional connectivity is improved by long scan length (Braun et al., 2012; Birn et al., 2013). It is worth investigating the influence of scan length on the ICC of DS/DN. Lastly, there is the potential influence of scan sessions occurring at midnight, since we used the Midnight Scan Club dataset. Hill and Smith have examined the effect of time of day on the relationship between mood state, anaerobic power, and capacity, and they found that the relationship between mood state and subsequent performance is dynamic and is dependent upon the time of day (Hill et al., 1993). This study identified the whole-brain distribution of DS/DN in resting-state fMRI; however, it remains unclear whether DS/DN is more associated with neuronal activity or non-neuronal noises such as head motion. With the quantitative measures reported in the current study, more research is needed to further explore the mechanism underlying DS/DN in relation to fMRI preprocessing and the underlying functional connectivity.

## Conclusion

In this paper, we investigated the degree of stationarity (DS) and the degree of non-linearity (DN) of rs-fMRI time series of all gray matter voxels and intrinsic brain networks from the Midnight Scan Club datasets. Results from this study suggest that the baseline signals from the VN, LIMB, SMN, and DMN have relatively greater non-stationarity and non-linearity compared with those of the VAN, DAN, and FPN. This suggests that when we compute the “static” functional connectivity, it is necessary to take into account the relative contribution from the non-linearity and non-stationarity components from the respective brain regions. For example, when analyzing static functional connectivity, the VN needs to have more non-linear and non-stationary components eliminated than does the FPN. Moreover, the VN, LIMB, and DMN networks were more non-linear and non-stationary, so shorter-time data can be used, because the shorter the time, the closer the characteristics of the data are to being stationary and linear, so the optimal length of time is required. If the non-stationary and non-linear properties are not considered, then the results will be an approximate phenomenological description of the real characteristics. Our results suggest that different functional networks have distinct non-stationarity and non-linearity owing to the complexity and stability of rs-fMRI time series. Moreover, the DS and DN measures not only enable the characterization of the brain's regional signal properties but also of the individual subject's brain dynamic features. Therefore, this quantitative DS/DN method provides a tool for future research to further explore the effect of DS/DN on fMRI measures such as functional connectivity and to improve neural activity extraction or simulation by considering non-linear and non-stationary components.

## Data Availability Statement

Publicly available datasets were analyzed in this study. This data can be found here: the Midnight Scan Club data (https://openfmri.org).

## Author Contributions

SG made substantial contributions to the conception and design of the work, analysis, interpretation of data for the work, and the draft of the manuscript. RJ and HB made substantial contributions to the preprocessing analysis and interpretation of data for the work. JY and PX made a contribution to the revision of the manuscript. BB made a contribution to the conception and design of the work. As the corresponding author, BB and CM made great contributions to the interpretation of fMRI data and determined the final version to be published. All authors have read and approved the final manuscript.

## Conflict of Interest

The authors declare that the research was conducted in the absence of any commercial or financial relationships that could be construed as a potential conflict of interest.
